# Physical Activity in Schizophrenia is Higher in the First Episode than in Subsequent Ones

**DOI:** 10.3389/fpsyt.2014.00191

**Published:** 2015-01-05

**Authors:** Sebastian Walther, Katharina Stegmayer, Helge Horn, Nadja Razavi, Thomas J. Müller, Werner Strik

**Affiliations:** ^1^University Hospital of Psychiatry, Bern, Switzerland

**Keywords:** actigraphy, antipsychotic, negative symptoms, hypokinesia, psychosis

## Abstract

Schizophrenia is frequently associated with abnormal motor behavior, particularly hypokinesia. The course of the illness tends to deteriorate in the first years. We aimed to assess gross motor activity in patients with a first episode (*n* = 33) and multiple episodes (*n* = 115) of schizophrenia spectrum disorders using wrist actigraphy. First episode patients were younger, had higher motor activity and reduced negative symptom severity. Covarying for age, chlorpromazine equivalents, and negative symptoms, first episode patients still had higher motor activity. This was also true after excluding patients with schizophreniform disorder from the analyses. In first episode patients, but not in patients with multiple episodes, motor activity was correlated with antipsychotic dosage. In conclusion, after controlling for variables related to disorder chronicity, patients with first episodes were still more active than patients with multiple episodes. Thus, reduced motor activity is a marker of deterioration in the course of schizophrenia spectrum disorders.

## Introduction

The first episode is of particular interest to schizophrenia research because it allows assessing the clinical presentation in the absence of factors related to illness chronicity. Longitudinal studies have demonstrated a strong decline in function and quality of life during the first 2–5 years. Despite vast heterogeneity in illness course, the condition during initial episode has some predictive value (1).

Motor abnormalities are intrinsic to schizophrenia and have been reported throughout the whole course of the disorder irrespective of medication status (2–6). Particularly, psychomotor slowing impacts cognitive performance and outcome (7). Objective measures acquired with actigraphy have established hypokinesia in schizophrenia, which is correlated with negative symptom severity (8–10). However, objectively assessed gross motor activity has not been tested in first episode patients. Even though 67% of unmedicated first episode patients present with at least one motor sign (11), first episode patients tend to present with less or comparably severe negative symptoms as patients with multiple episodes (12, 13). Thus, the current study aimed to test whether physical activity as measured by wrist actigraphy would differ between first episode and multiple episode patients with schizophrenia spectrum disorders. Furthermore, we aimed to explore the association of physical activity with antipsychotic dosage and negative syndrome severity in both groups. We hypothesized that physical activity was higher in first episode patients compared to multiple episode patients, as functional decline often occurs within the early course of the disorder (1).

## Materials and Methods

### Patients

To explore differences between patients with a first vs. multiple episodes on physical activity, we pooled data from previous and ongoing studies applying actigraphy in schizophrenia spectrum disorders (9, 14–19). A total of 148 patients with schizophrenia spectrum disorders were included (first episode *n* = 33, multiple episodes *n* = 115). The patients with multiple episodes had on average 7.9 episodes (SD = 6.2). Diagnoses were given according to DSM-IV criteria after thorough clinical examination and review of all case files by board certified psychiatrists. The local mental health care system ensures that most of the patients are admitted to our clinic in every psychotic episode requiring inpatient treatment, allowing the collection of reliable diagnostic information. In a proportion of studies that contributed data to this analysis, the diagnoses were ascertained by structured clinical interviews such as SCID and MINI (27% of cases). Diagnoses given included paranoid schizophrenia (33% of first episode vs. 55% of multiple episode patients), catatonic schizophrenia (12 vs. 11%), disorganized schizophrenia (0 vs. 16%), schizoaffective disorder (6 vs. 5%), and schizophreniform disorder (49 vs. 13%) with significant differences between groups (χ^2^ = 21.7, df = 1, *p* < 0.001). Patients were excluded in case of substance abuse other than nicotine, medical conditions affecting physical activity, and epilepsy. Physical activity was recorded approximately 2 weeks after admission to the psychiatric department. At the same time, psychopathology was assessed using the positive and negative syndrome scale (PANSS) (20). Most patients were medicated (71% received atypical antipsychotics, 18% received mixed medication of typical and atypical antipsychotics, 6% received only typical antipsychotics, and 5% were medication-free at the time of our assessments). Chlorpromazine equivalents (CPZ) of the current antipsychotic pharmacotherapy were calculated according to Woods (21). In addition, diazepam equivalents (DE) were calculated according to Ashton (22). The protocols of the studies applying wrist actigraphy in schizophrenia spectrum disorders had been approved by the local ethics committee and participants provided written informed consent prior to study inclusion.

### Actigraphy

Participants wore an actigraph (Actiwatch, Cambridge Neurotechnology, Inc., Cambridge, UK) for 24 consecutive hours at the wrist of the non-dominant arm. The piezoelectric sensor converts acceleration into movement counts. Data were sampled in 2 s intervals. Participants provided sleep log information and information on recording pauses (due to showering or bathing). Only the data collected during wakeful periods of the 24 h recording time were analyzed. Activity counts were averaged to provide the activity level (AL) in counts/h.

### Statistical analyses

Demographic and clinical parameters were compared between groups by one-way ANOVAs and χ^2^-tests. Since groups differed in the PANSS negative syndrome subscale score, age, and CPZ dosage, these variables were entered as covariates in an ANCOVA of AL. Furthermore, as the group of first episode patients included a large proportion of patients with schizophreniform disorder, we recalculated the ANCOVA excluding all patients with schizophreniform disorder. In order to test the additional effects of illness duration and benzodiazepine administration, we recalculated the ANCOVA first including duration of illness, negative symptoms, and CPZ as covariate and second including duration of illness, negative symptoms, CPZ, and DE as covariates. Furthermore, we compared AL in patients with different medication types (typical antipsychotics, atypical antipsychotics, and mixed medication). Next, clinical and demographic variables were correlated with AL for both groups separately. Because of the divergent results, we also calculated the interaction of CPZ and group on AL using an ANCOVA in the whole sample. Besides the classical PANSS subscores, we also tested the five PANSS factors according to van der Gaag and colleagues (23) (positive, negative, disorganization, excitement, and emotional distress), and the avolition score (sum of items N2 + N4) according to Bervoets et al. (24). All tests were performed with SPSS 21.

## Results

Gender distribution was not different between groups (first episode patients 61% male, multiple episode patients 57% male, χ^2^ = 0.175, df = 1, *p* = 0.696).

First episode patients had increased AL, were younger, received lower doses of antipsychotics and had a trend to reduced PANSS negative scores (see Table 1). Furthermore, first episode patients had reduced scores in the PANSS factors negative, disorganization, and excitement (23), as well as the PANSS avolition score (24).

**Table 1:** **Clinical and demographic characteristics**.

	First episode patients (*n* = 33)	Multiple episode patients (*n* = 115)	*F*-value	*p*-Value
Activity level (counts/h)	18057 (9194)	13551 (7047)	9.7	0.002
Age (years)	31.9 (12.5)	39.6 (11.2)	15.4	<0.001
Duration of illness (years)	1.7 (3.2)	12.7 (10.2)	30.9	<0.001
CPZ (mg)	360.2 (341.2)	530.5 (399.9)	3.9	0.049
DE (mg)	6.3 (10.5)	5.0 (10.1)	0.6	0.547
PANSS positive subscale	13.4 (4.6)	15.7 (5.6)	1.5	0.226
PANSS negative subscale	15.9 (6.5)	18.1 (6.2)	3.2	0.078
PANSS total score	61.1 (15.8)	68.7 (16.2)	5.5	0.020
PANSS avolition	4.0 (2.2)	5.0 (2.2)	5.3	0.022
PANSS factor positive	16.1 (7.1)	17.7 (6.6)	1.4	0.239
PANSS factor negative	15.2 (7.8)	18.3 (7.0)	4.6	0.033
PANSS factor disorganization	20.9 (5.7)	24.2 (6.6)	6.6	0.011
PANSS factor excitement	13.2 (3.9)	15.4 (5.3)	5.1	0.026
PANSS factor emotional distress	14.7 (6.1)	16.0 (5.5)	1.4	0.242

*Numbers are given as mean (SD)*.

The difference in AL between groups remained when adding the PANSS negative syndrome subscale score, age, and CPZ as covariates (*F* = 6.2, df = 4, *p* < 0.001, η^2^ =0.15). Furthermore, including duration of illness, negative symptoms, and CPZ as covariate did not substantially alter the results (*F* = 4.53; df = 4; *p* = 0.002; η = 0.12). Moreover, differences in AL between groups remained stable with the inclusion of duration of illness, negative symptoms, CPZ, and DE as covariates (*F* = 4.23; df = 5; *p* = 0.001; η = 0.13). Likewise, the difference in AL between groups remained when all subjects with schizophreniform disorder were excluded from the ANCOVA (*F* = 4.2, df = 4, *p* = 0.003, η^2^ = 0.13) with the PANSS negative syndrome subscale score, age, and CPZ as covariates.

Finally, AL did not differ between groups with distinct antipsychotic medication (typical, atypical antipsychotics, and mixed medication) (*F* = 0.96; df = 2; *p* = 0.386; η = 0.01).

Correlations between AL and clinical parameters are given in Table 2. Associations with measures of the negative syndrome were more dominant in the patients with multiple episodes. Groups differed in the correlation of CPZ and AL, with significant effects in first episode patients and no effects in patients with multiple episodes (ANCOVA of AL with intercept between CPZ and group *F* = 11.3, df = 1, *p* < 0.001, η^2^ = 0.07).

**Table 2:** **Correlation between activity level and clinical parameters**.

	First episode patients		Multiple episode patients	
	*r*	*p*	*r*	*p*
Age	−0.21	0.251	−0.19	0.038
CPZ	−0.46	0.008	0.08	0.411
DE	−0.03	0.893	−0.29	0.002
PANSS positive syndrome score	0.07	0.698	0.13	0.163
PANSS negative syndrome score	−0.02	0.910	−0.29	0.001
PANSS total score	−0.07	0.708	−0.17	0.070
PANSS avolition factor	−0.38	0.031	−0.29	0.002
PANSS positive factor	−0.05	0.784	−0.01	0.897
PANSS negative factor	−0.33	0.064	−0.32	<0.001
PANSS disorganization factor	0.32	0.073	−0.08	0.399
PANSS excitement factor	−0.04	0.835	−0.08	0.418
PANSS emotional distress factor	−0.05	0.800	−0.13	0.183

## Discussion

In the present study, we were able to demonstrate that patients in a first episode of schizophrenia spectrum disorders have higher physical activity than patients with multiple episodes. This result held true after controlling for negative symptoms, age, dosage of antipsychotics, and administration of benzodiazepines. Furthermore, it was still evident after excluding patients with schizophreniform disorder. Thus, we are confident that there is a true difference in physical activity between the groups beyond other major differences that are related to illness duration. Of course, the magnitude of the effect was small to medium in size as there are also other factors contributing to physical activity besides age, chronicity, and negative symptoms, such as lifestyle habits and physical constitution. Still, the mean AL value of the first episode patients is close to the mean of AL reported in different samples of healthy controls, e.g., 18000–21000 counts/h (8, 15, 25). Furthermore, subjects at ultra high risk for psychosis were shown to have reduced ALs at trend level compared to matched controls (26). Thus, even though the AL of first episode patients might still be slightly reduced compared to age matched controls, AL appear comparable to those of healthy control groups with wider age ranges. Our finding is in line with the observation that motor abnormalities are present from the first episode of schizophrenia spectrum disorders but tend to deteriorate with the progression of the disorder (2, 5). Likewise, neurocognitive measures of fine motor performance have indicated impairments in first episode patients and even more in chronic patients (27). However, it remains unknown, which factors drive the reduction of physical activity in patients with multiple episodes. Clearly, negative symptoms are related to reduced physical activity (8–10). In addition, the use of catatonia rating scales such as the modified Rogers scale (28) indicated that the frequency of the item “marked underactivity” was increased in a mixed sample of schizophrenia spectrum disorder patients as compared to first episode patients (28 vs. 9%) (11, 29). In general, motor abnormalities are prevalent in the early course but increase in frequency with multiple episodes. In many instances, it is hard to disentangle the multiple causes of hypokinesia, such as catatonia, parkinsonism, or reduced volition, i.e., negative symptoms (2). If reduction of physical activity was an indicator of deterioration, it would well fit in the observation of clinical deterioration within the first 2–5 years after the onset of schizophrenia spectrum disorders (1). First evidence suggests that hypokinesia due to parkinsonism in first episode patients was predictive of poor cognitive function after 6 months (30). However, schizophrenia presents with a heterogeneous longitudinal course and deterioration of symptoms is not everyone’s fate (31).

Another important finding of the present study is the interaction of antipsychotic dosage and group on AL. Whereas, in multiple episode patients, we were unable to find a correlation of AL and CPZ, increased antipsychotic dosage was related to lower AL in first episode patients. Here, we may speculate that in first episode patients in the absence of marked negative symptoms, parkinsonism, or catatonia, antipsychotic agents may exert more impact on the overall motor activity. However, this view is partly challenged by the fact that antipsychotic agents ameliorate most psychomotor impairments in previously naïve first episode patients, including hypokinesia, parkinsonism, and catatonia (11). Still, the various antipsychotic agents currently used may exert differential effects on physical activity or spontaneous, i.e., preexisting motor symptoms. So far, only few reports exist investigating the effect of different antipsychotic agents on motor behavior in previously unmedicated subjects (32). However, we found no differences of AL in patients with different medication types (typical, atypical antipsychotics, and mixed medication).

In line with previous reports, reduced motor activity was associated with negative symptoms in both first episode and chronic schizophrenia spectrum disorders (8, 9). Also, reduced tongue movements were demonstrated to correlate with negative symptoms in first episode patients (33). Likewise, hypokinesia was associated with negative symptoms in first episode patients (11). In chronic schizophrenia, various measures of hypokinesia have demonstrated correlations with negative symptom severity, particularly the initiation of movements seems affected (2, 24, 29, 34, 35). As in one previous report, the avolition component of the negative syndrome displayed strongest correlations with spontaneous motor activity (8). Thus, our data support the assumption that actigraphy may particularly well suited to assess motivational aspects of the negative syndrome. Finally, exercise interventions have regained interest in schizophrenia targeting various problems of these patients, such as metabolic, cognitive, and social problems (36). First controlled studies reported positive effects of exercise interventions on negative symptoms and working memory in schizophrenia (37).

This study has some limitations requiring discussion. We have used data from a database to answer the research question. The methods of actigraphy and PANSS assessment were the common ground. If we had to conduct a new study on this topic, we would include more standardized measures of motor function focusing on parkinsonism and catatonia. Parkinsonism and catatonia may have influenced AL (2, 38). However, both symptoms are frequently present in patients with a first psychotic episode and in patients with multiple episodes (11). Still, these symptoms might be more frequent in patients with a chronic course of the disease (14). However, treatment with antipsychotics shows a heterogeneous effect on parkinsonism and catatonia in previously antipsychotic naïve patients: in some patients, these symptoms are ameliorated by treatment, in others they tend to emerge or deteriorate during pharmacotherapy, while in a third group symptoms remain unchanged with antipsychotic medication (32). In addition, there are better scales to assess negative symptoms (39). Still, the PANSS is a commonly used instrument, which aids data interpretation. Finally, we did not measure depressive symptoms, even though, depression might affect ALs. Patients with major depression move less than healthy controls (40). Still, in a previous investigation, we found only weak associations in major depressed patients between depression severity as assessed by the Hamilton depression rating scale (HAMD) and AL (41). However, taking the emotional dysregulation factor according to van der Gaag (23) as a substitute parameter for depression, we failed to identify correlations with AL in any of the two groups.

In conclusion, we demonstrated that first episode patients have higher physical activity than patients with multiple episodes. The finding underlines that functional deterioration including reduced motor activity frequently occurs beyond the first episode. Thus, future studies should evaluate whether this reduction may be targeted by pharmacotherapy or specialized programs to promote physical exercise in schizophrenia (36, 42).

## Author Contributions

Dr. Sebastian Walther and Dr. Helge Horn designed the study. Dr. Sebastian Walther wrote the protocol. Drs. Nadja Razavi, Katharina Stegmayer, and Sebastian Walther recruited participants and performed assessments. Dr. Sebastian Walther performed the data analyses. All authors interpreted and discussed the findings. Dr. Sebastian Walther wrote the first draft of the manuscript. All authors contributed to the final version of the manuscript.

## Conflict of Interest Statement

The authors declare that the research was conducted in the absence of any commercial or financial relationships that could be construed as a potential conflict of interest.
